# Global Health Policy and Access to Care: Investigating Patient Choice on an International Level Using Social Media

**DOI:** 10.3389/fpubh.2015.00284

**Published:** 2016-01-25

**Authors:** Peter Zhukovsky, Kai Ruggeri, Eduardo Garcia-Garzon, Sara Plakolm, Elisa Haller, Dafina Petrova, Vaishali Mahalingam, Igor G. Menezes

**Affiliations:** ^1^Department of Psychology, University of Cambridge, Cambridge, UK; ^2^Policy Research Group, Department of Psychology, University of Cambridge, Cambridge, UK; ^3^Department of Engineering, Engineering Design Centre, University of Cambridge, Cambridge, UK; ^4^Departamento de Psicología Social y Metodología, Facultad de Psicología, Universidad Autónoma de Madrid, Madrid, Spain; ^5^Unit for Paediatric and Adolescent Psychiatry, Division of Paediatrics, University Medical Centre Maribor, Maribor, Slovenia; ^6^Clinical Psychology with Focus on Psychotherapy Research, Department of Psychology, University of Zurich, Zürich, Switzerland; ^7^Mind, Brain, and Behavior Research Center, University of Granada, Granada, Spain; ^8^The Psychometrics Centre, University of Cambridge, Cambridge, UK; ^9^Quantitative Methods and Predictive Psychometrics Laboratory, Institute of Psychology, Federal University of Bahia, Salvador, Brazil

**Keywords:** medical travel, public health policy, decision-making, destination, cost, quality, factors

## Abstract

**Background:**

Increased access to transportation and information has led to the emergence of more diverse patient choice and new forms of health care consumption, such as medical travel. In order for health care providers to effectively attract patients, more knowledge is needed on the mechanisms underlying decision-making of potential travelers from different countries. A particularly promising method of studying the travelers’ motives is collecting data on social media.

**Objectives:**

The aim of this study was to test what factors influence decision-making of potential medical travelers and how these factors interact. Based on existing literature, the factors analyzed included quality, cost, and waiting time for 2 procedures varying in invasiveness across 12 different destination countries.

**Methods:**

Decision-making patterns were examined using a pilot questionnaire that generated a large amount of data from over 800 participants in 40 countries. Participants indicated their willingness to travel given different scenarios. Each scenario consisted of a combination of several factors. Additionally, participants were asked to indicate the reasons for their choice.

**Results:**

Individuals display high willingness to travel for medical care when combining all participants and scenarios, travel for care was chosen 66.9% of the time. Among the factors influencing their decisions, quality of the medical procedure abroad was considered most important, and cost was least important as shown by chi-square tests and corresponding odds ratios. Log-linear analyses revealed an interaction between time waiting in the local health care system and type of procedure, whereby time pressure increased the odds of agreeing to travel for the more invasive procedure. The odds of traveling to Europe and the USA were by far the highest, although participants indicated that under certain conditions they might be willing to travel to other medical destinations, such as Asia.

**Conclusion:**

Our measurements yielded several reliable insights into the factors driving medical decision-making. An essential next step would be to expand these findings with a more encompassing sample and more elaborate statistical modeling.

## Introduction

National health care systems differ largely in terms of structure, provision of services, quality, and costs. These differences, combined with increased access to transportation across borders, and the availability of information brought on by technological advances, are leading to the emergence of patient choice, new forms of consumerism, and production of health care services (1). One dimension of this development is the selective movement of patients beyond national borders to pursue medical treatment, a phenomenon that has been labeled as “medical tourism” or “medical travel” (2).

Though medical travel itself is not new, there has been a shift in travel patterns in recent years (2). Wealthy individuals traveling abroad to obtain more advanced health care and better quality treatment are no longer representative of the situation (3). Instead, a rising number of individuals from developed countries travel to developing countries for the same purposes. Many of them cannot be characterized as affluent individuals but rather as conscious consumers seeking affordable high-quality medical care (4). In this emerging context, medical travel is defined as any patient crossing national borders with the purpose of receiving treatment that has been determined as essential to maintain quality of life by a health professional but may not need to be performed urgently.

Estimated numbers of patients traveling abroad for medical care vary (5), but all of them indicate an immense growth in this phenomenon (4, 6). Despite the expansion of a global health care industry and considerable attention from researchers, policy makers, and the media, hard evidence-based reports on the flow of medical travelers are absent. Although initial estimates were based on a limited body of empirical evidence, they were cited so often that they became treated as absolute. However, it is now recognized that they should be interpreted with caution (6, 7).

Moreover, the majority of existing literature on medical travel focused on medical tourism [e.g., Ref. (8, 9)], more specifically on traveling for elective procedures, such as cosmetic surgery (2), as opposed to traveling for necessary medical procedures [e.g., Ref. (10, 11)]. Only a small number of studies have focused on identifying push factors that make a person elect to travel for medical care and pull factors that cause a person to select a particular type of treatment (12–14). To date, these factors include treatment accessibility in the home country and the nature of the treatment, commonly orthopedic, eye, and heart surgery (3). Based on the limited available evidence (15, 16), the primary reasons for seeking medical treatment abroad include the prospect of access to higher-quality treatment, lower costs, and shorter waiting lists (7, 10, 17–19).

Although current literature has been helpful in identifying some key factors in decision-making process when it comes to traveling for medical care, it is not clear which factors have more influence and whether they interact with each other to influence decision-making. To address this lack of evidence (20), we investigated which factors influence individuals’ decision for or against obtaining medical care abroad the most and how these factors interact with each other. The factors included the location of the destination clinic, waiting time, cost, quality, and invasiveness of the procedure. A survey, covering a large number of hypothetical medical travel decisions constructed as combinations of the factors mentioned above, was used to elucidate the structure of decision-making of potential medical travelers from various geographical regions.

## Materials and Methods

### Study Design

A binary (Yes/No) questionnaire was developed in order to assess under which circumstances people would decide in favor or against medical travel. The design of the present questionnaire was developed similarly to the measurement used in the London Patient Choice Project (LPCP) and is explained in more detail by Garcia-Garzon and colleagues (Garcia-Garzon et al., under review). The LPCP investigated the factors influencing patients’ socioeconomic choices using a combination of revealed and stated preferences (17).

**Figure 1:**
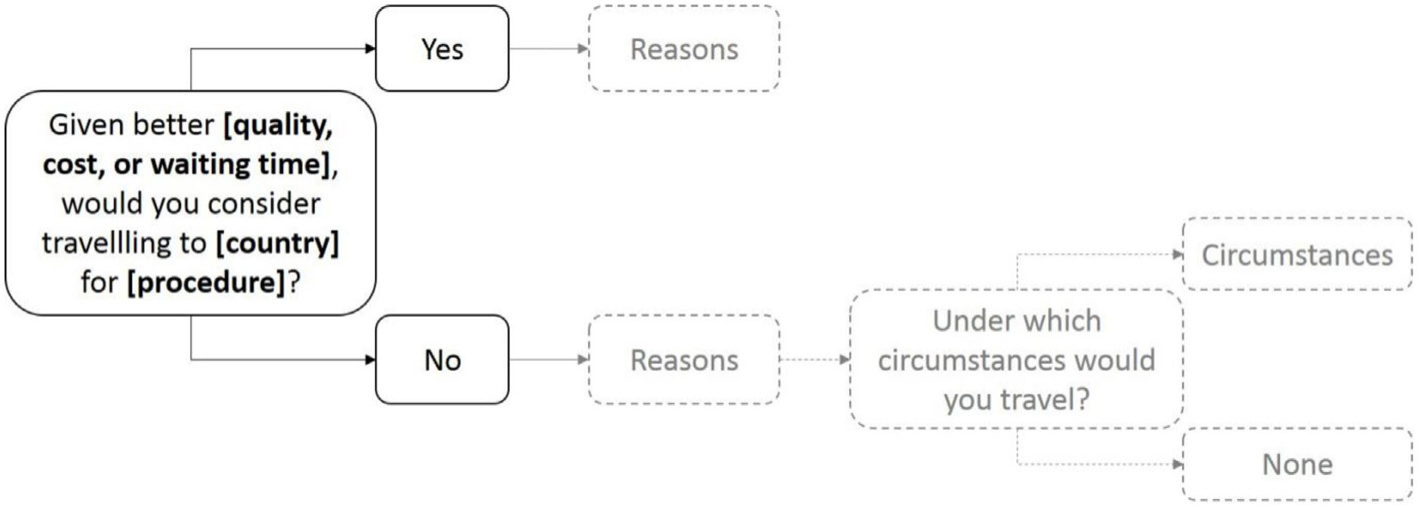
**Structure of the questionnaire scenarios**. After stating their decision to travel for care, participants were asked to tick reasons relevant to their decision (in gradient).

The present questionnaire consisted of hypothetical scenarios framed in a realistic way. The scenarios required respondents to imagine being in need of a specific medical treatment given a certain initial situation. The hypothetical scenarios were a combination of three different factors: procedure, reason for traveling, and the country of the destination clinic (Figure 1). Two specific procedures were selected from a pool commonly referred to in medical travel literature [e.g., Ref. (21)] and aimed to capture two distinct levels of invasiveness, whereby “hip replacement” is considered to be a less invasive and less life-threatening procedure than “heart valve replacement.” Invasiveness was considered important as it should influence the perceived urgency of the treatment. The more invasive procedure is assumed to be perceived as more threatening and hence more urgent. Three reasons for traveling – quality, waiting time, and cost – were included in the questionnaire based on a literature review and consultancy expertise that implicated these factors in determining patients’ decisions to receive care abroad. Finally, 12 countries were selected based on their prospective attractiveness for medical travelers. For the purpose of the analyses, these countries were grouped into six regions: Northern Europe (Germany and UK), Southern Europe (Portugal and Malta), Middle East (Qatar and Dubai), Southeast Asia (Thailand and Philippines), Pacific (Singapore, New Zealand, and Australia), and the USA as its own region. Each scenario specified one out of the two procedures, an advantage in cost, waiting time, or quality and was set in one of the 12 destination countries. The dependent variable was the participants’ choice to receive care abroad for each scenario.

The permuted combinations of 12 countries, two procedures and three factors resulted in a pool of 72 scenarios. Besides the demographic items, such as rating of the aspects of local health care and socioeconomic status details, each participant was presented with a random selection of six scenarios. By doing this, a lengthy questionnaire was avoided and a multiplicity of observations was made possible to be obtained. This approach was crucial for another reason: randomizing the scenarios increased the independence of observations and decreased the systematic variation in the responses that would otherwise arise from a fixed order of scenarios.

### Sample

Participants were recruited via social media advertising and completed the questionnaire in English online. Data were excluded if participants only completed a fraction of the questionnaire and did not answer the questions for all six scenarios or took too much or too little time to complete the questionnaire. Valid data were obtained from 543 participants, resulting in 3155 observations. The majority of the sample were female (67.8%), highly educated (74.5%), and European (87.7%). On average, it took participants a little more than 7 min to complete the questionnaire. Ethical approval was obtained from the Department of Engineering Ethics Committee at the University of Cambridge.

### Statistical Analysis

Pearson’s Chi-Square tests with contingency tables were used to analyze the relationship between the main predictors or demographic variables with the dependent variable (choice to travel for medical care). To extend the analyses to interaction effects of procedure, reason, and destination region on the choice to receive care abroad, multiple logistic regression was used.

First, chi-square tests and linear regression were used to test for associations between demographic control variables and the frequency of agreeing to travel for care. For the purposes of categorical analyses, Likert scale ratings (1–5) and the destination regions were treated as categories (22). Second, goodness-of-fit chi-square test was used to test whether there was an overall tendency to agree to travel for medical care. Next, a chi-square test was used to test for associations between the procedure and the indicated choice to travel. The effect was quantified in terms of the ratio of the odds of traveling for heart valve and the odds of traveling for hip replacement (23, 30). Similar tests were run to test for associations between reason or destination region with the stated choice to travel. Furthermore, frequencies of the reasons indicated as important for the stated decision to travel in a given hypothetical scenario were recorded for the most interesting scenarios with unusual response patterns. Specifically, since participants were not likely to choose to travel for care to Asia, it was important to analyze post-decision information for that region to clarify the motivation behind negative decisions. In order to clarify the interactions between the predictor variables, log-linear analyses were carried out. In particular, interactions between main predictors, such as procedure and reason, were included alongside with interactions between control variables, such as rating of health care aspects in one’s home country, and the reason presented in the scenario.

## Results

### Descriptives

On average, participants rated the quality and the cost of their local health care system positive to very positive, and assigned a neutral to below neutral rating to the waiting time in their local health care system (see Table 1). A small proportion of participants (5%) had previous medical travel experience. Overall, two-thirds of the responses indicated willingness to travel for medical care, when main predictor variables were not taken into account. Most participants reported that their income was average compared to the income of a fully employed person in the same country.

**Table 1:** **Characteristics of study sample (*N* = 543)**.

Variable	Value	Frequency % (absolute Nr)	Mean	SD
Gender				
	Female	67.8 (368)		
Age			27.6	10.36
Medical travel experience				
	Yes	5.0 (27)		
	No	95.0 (516)		
Region of origin				
	Europe^a^	87.7 (470)		
	Other	12.3 (73)		
Education				
	Master degree, PhD or eq.	43.6 (237)		
	Bachelor degree or eq.	30.9 (168)		
	Secondary school	19.0 (103)		
	Higher vocational training	6.5 (35)		
Income (*n* = 542)				
	Very high	1.7 (9)		
	Above average	22.1 (120)		
	Average	43.5 (236)		
	Low	29.8 (162)		
	Below poverty	2.8 (15)		
Rating of local health care^b^				
	Quality		3.7	0.98
	Waiting		2.7	1.13
	Cost		3.5	1.11

*^a^Europe, Germany, Slovenia, UK, Spain, and other countries*.

*^b^Values refer to a Likert scale ranging from 1 to 5, with 1, very negative; 2, negative; 3, neutral; 4, positive; 5, very positive*.

### Stated Choice to Travel by Control Variables (Demographics)

Chi-square tests were conducted to test for associations between control variables and the decision to travel or not to travel for care (see Table 2).

**Table 2:** **Chi-square statistics for control variables**.

Variable	df	χ^2^
Gender	1	3.04
Income	4	11.12*
Education	3	19.49**
Ratings of aspects of local health care		
Cost	4	8.65
Quality	4	41.24**
Waiting time	4	20.04**

*p < 0.05.

**p < 0.01.

Participants who rated waiting time as more problematic in their own country were more likely to opt for medical travel. The linear regression model reveals a negative trend between the rating of the local health care waiting time and the choice to travel for care, operationalized as the odds of traveling [*F*(1,3) = 68.45, *p* < 0.005] with an adjusted *R*^2^ of 0.94. Participants who rate waiting time as very negative are twice as likely to agree to travel as those who rate it as very positive, OR = 2.04 with 95% CI (1.39, 3.00).

No significant correlations were found between the odds of agreeing to medical travel and the rating of quality and between odds of traveling [*F*(1,3) = 3.88, *r* = −0.75, *p* > 0.05 and *F*(1,3) = 3.68, *r* = −0.74, *p* > 0.05, respectively].

### Stated Choices to Travel for Medical Care

**Figure 2:**
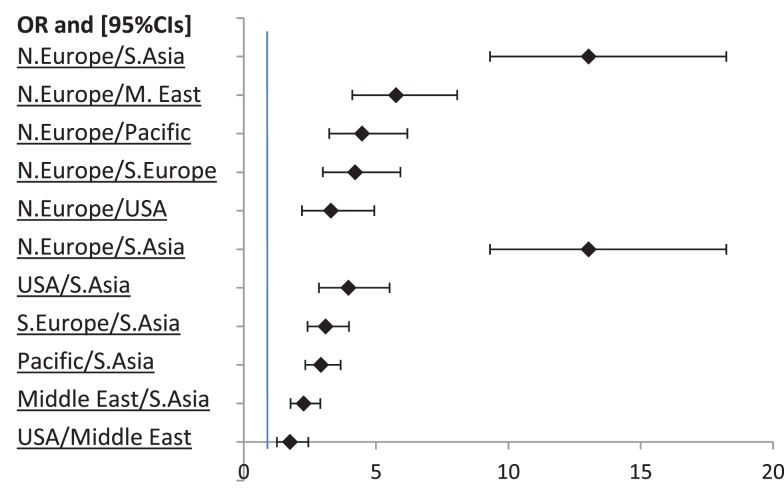
**Odds ratios for Northern Europe (N. Europe), Southeast Asia (S. Asia), and Middle East (M. East) with 95% CIs**. Non-significant ORs are not displayed.

Without controlling for other factors, in most of the 3155 scenarios (66.9%) that were presented, participants agreed to travel for care, χ^2^ (1) = 362.29, *p* < 0.001 (Table 3). A significant association was found between the procedure and the choice to travel for medical care, χ^2^ (1) = 12.23, *p* < 0.001. Participants in the heart valve scenario were more likely to travel for care than participants presented with hip replacement, which represents a small effect of the medical procedure. There was a significant association between the reasons for medical travel and the decision to travel for care: χ^2^ (2) = 180.16, *p* < 0.001. Participants were most likely to travel for quality, followed by waiting time and cost. In addition, there is a significant association between the destination and the decision to travel for medical care: χ^2^ (5) = 295.51, *p* < 0.001. Germany or UK (Northern Europe) are the most likely destinations, with the odds of agreeing to travel to these countries being over three times as high as the odds of traveling to any other region (see Figure 2). Participants were less likely to travel to Thailand and Philippines (Southeast Asia, odds = 0.72), as the odds of other regions, including Southern Europe, Northern Europe, USA, and Pacific were at least two times as high as the odds of traveling to Southeast Asia (see Figure 2).

**Table 3:** **Frequency of deciding for or against medical travel and the odds ratios of traveling by reason and procedure**.

Across all scenarios	(%)		OR (95% CI)
Choice to travel (circumstances not controlled)	66.9		
By procedure			
Hip replacement	64.0	Heart valve	1.3 (1.1; 1.6)
Heart valve	69.8	Hip replacement	
By reason			
Quality	82.0	Quality/waiting time	2.6 (2.1; 3.3)
Waiting time	63.5	Quality/cost	3.7 (3.0; 4.5)
Cost	55.4	Waiting time/cost	1.4 (1.2; 1.6)

The odds of traveling to the Middle East were the second lowest (odds = 1.63), being 1.75 times lower than the odds of agreeing to travel to the USA, and 5.75 times lower than the odds of UK and Germany (Figure 2). The odds of traveling for care were higher than 1 for all the regions of destination except Southeast Asia, indicating that the number of “yes” replies for those regions was higher than the number of “no” replies. For Southeast Asia, however, the odds of traveling for care were lower than 1, indicating that the likelihood of a participant not willing to travel to this region was higher than the likelihood of agreeing to travel.

**Figure 3:**
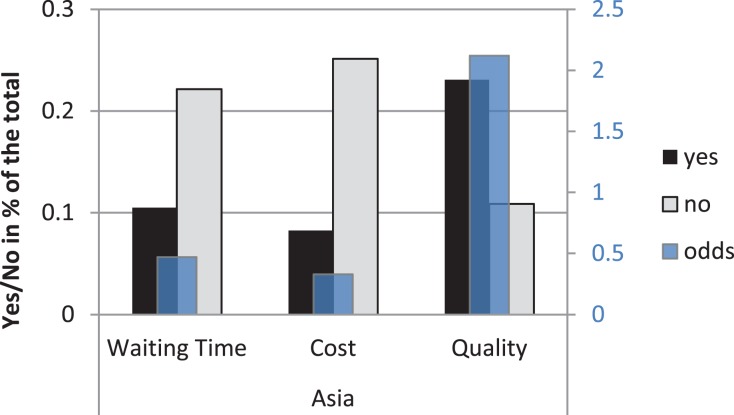
**Choice to travel (Yes/No frequencies in percent of the total) to Southeast Asia by reason on the left *y*-axis and odds of traveling on the right *y*-axis**.

Since the stated preference rates for medical travel were lowest for Southeast Asia, this region provides some crucial insights into factors driving medical traveler’s decisions to go abroad and deserves further analysis. A breakdown of the willingness to travel to that region by reason (Figure 3, the left *y*-axis) shows that the willingness to travel differs significantly across the three reasons when participants are presented with Southeast Asia as destination of medical travel, χ^2^ (2) = 78.84, *p* < 0.001. The decision to travel is significantly more frequent if participants are told they would receive higher quality of treatment than if they are presented with shorter waiting times or lower costs in Asia. To quantify this effect, the odds of traveling to Asia for quality (reported on the right *y*-axis in Figure 3) are 6.5, 95% CI (4.07, 10.25) times higher than the odds of traveling there for cost and 4.5, 95% CI (2.86, 6.98) times higher than the odds of traveling for waiting time.

**Figure 4:**
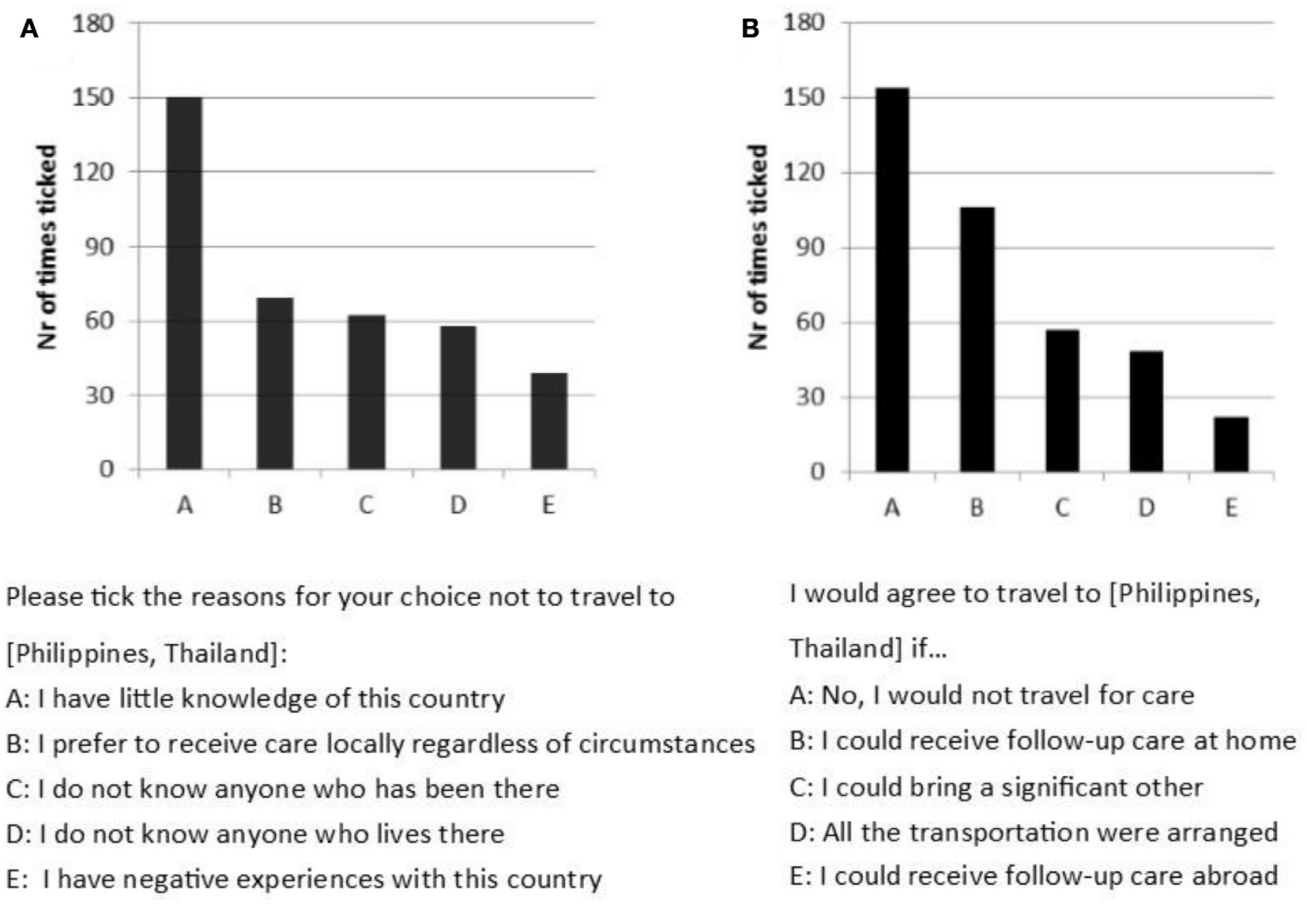
**(A)** Number of times a statement was indicated as important in deciding not to travel to Asia (maximum of 310 times possible). **(B)** Number of times a statement was indicated in response to “I would travel to Asia if …” (maximum of 310 possible).

Those who decided against traveling for care to Asia (310 negative replies) were presented with a choice of possible reasons that they could indicate as important for their previous decision. The most commonly indicated reason was that they had little knowledge of the country (see Figure 4A). When given the option to state their own reasons in an open question, participants pointed out that they had concerns about aspects of quality of care (20 cases) in Thailand or Philippines (such as hygiene), and that they would try to avoid the challenges of traveling so far away (17 cases) with a medical condition.

When asked whether they would reconsider their decision if they were given additional benefits, participants most often (in terms of most common response) indicated that they would not travel for care under any circumstances (see Figure 4B). However, in about a third of the cases, participants stated that they would travel if follow-up care in the home country was offered. In approximately 20% of the cases, participants were willing to travel if a significant other could accompany them. Several participants emphasized the importance of quality in the open questions as they agreed to travel for care if they would be provided with satisfactory assurance of high-quality treatment abroad, such as the approval by the local authorities or doctors.

### Stated Choice to Travel by Interaction between Reason and Procedure

The hypothesis that there are differences between the frequencies of agreeing to travel across combinations of different factors was tested using log-linear analyses. First, the interaction between reason and procedure was analyzed. The three-way log-linear analysis produced a final model that retained all effects. The model shows that the highest-order interaction was significant [reason × procedure × choice to travel, χ^2^ (1) = 16.59, *p* < 0.001]. To break down this effect, separate chi-square tests on the procedure and choice to travel for care were performed separately for cost, quality, and waiting time (reasons). A significant association between the procedure (heart valve and hip replacement) and waiting time was found, χ^2^ (1) = 27.79, *p* < 0.001.

**Figure 5:**
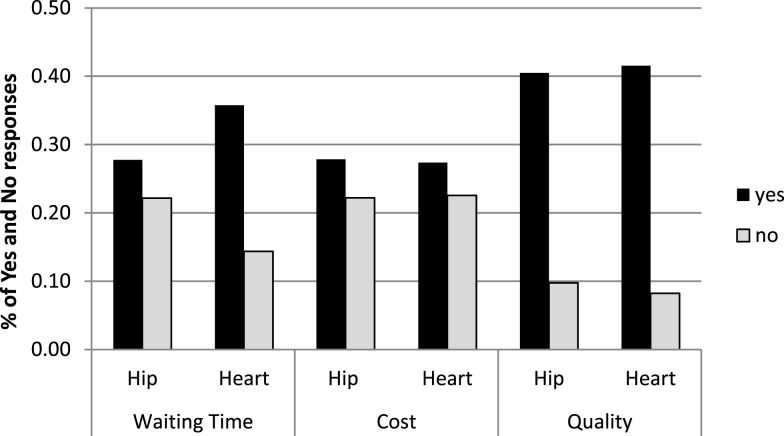
**Yes/no frequencies (in percent of the total) across procedure and reason**.

The odds of deciding in favor of medical travel in a hypothetical scenario with shorter waiting time were two times higher, 95% CI (1.54, 2.56) for heart valve than for hip replacement. In brief, it was found that while there are no interactive effects between procedure and cost or procedure and quality, the decision to travel given the waiting time scenario depends on the type of procedure. Reduced waiting time had a significantly stronger influence for the more invasive procedure, since heart valve replacement (in interaction with waiting time) was associated with higher odds of traveling for care (see Figure 5).

## Discussion

### Key Findings

Decision-making underlying medical travel is complex and influenced by a combination of factors. The objective of this study was to explore the contribution of certain factors that individuals consider when formulating their decision for or against traveling abroad for medical treatment. A new experimental tool was designed to test the relative influence of different destinations, procedures, reasons, and interactions of these factors on stated choices to travel for medical care.

Evidence emerging from this novel approach confirmed some of the findings in the previous literature and provided new insights into decision-making of non-patients, focusing on medical travel rather than medical tourism. Key findings included that (1) individuals display high willingness to travel for medical care; (2) quality is the most important and cost is the least important stated factor; (3) waiting time and procedure have interactive effects on decisions to go abroad for care; and (4) Northern Europe is exceptionally popular, followed by the USA and Southern Europe, though potential travelers would consider going to locations that were chosen less frequently, such as Asia, if certain conditions (notably assurance of higher quality) were provided. These findings were in line with the results of the control variable analysis.

### Control Variables

Although several associations exist between control variables and the stated willingness to travel exist, they do not provide evidence strong enough to be of interest. Indeed, they are much weaker than the associations between regions of destination and choice to travel as well as reason and the choice to travel. However, greater dissatisfaction with waiting times for local health care operationalized in the corresponding lower ratings reliably predicted higher odds of choosing to travel for medical care, as revealed by the regression analyses. Similarly, dissatisfaction with the quality and cost of local health care drives participants to go abroad for medical care, as shown by moderately strong correlation between the rating of quality or rating of cost and choice to travel.

Assuming that participants have the same priorities when receiving health care abroad and at home, this finding supports the result that quality and waiting time significantly influence participants’ decision-making. These factors influence their decision-making directly (as shown in the analysis of the hypothetical scenarios) and indirectly, when the participants are considering their local health care. Moreover, the answers of potential medical travelers in hypothetical scenarios were found to agree with the actual choices of medical travelers (London Patient Choice Report, 3). Since stated preference paradigm reliably indicated the revealed preference patterns, the conclusion that individuals value waiting time over cost can be generalized and holds even for expected revealed preference patterns.

### Individual Decisions for Medical Travel

Regardless of the combination of the motivating factors, participants were more likely to agree to travel for care than to refuse to travel. This confirms the trend that we find in the development of medical travel and tourism over the last decade: more and more people go abroad for medical care. Among UK citizens specifically, the number of medical travelers in 2010 was 6 times as large as that in 2000 (5). However, inferences about the willingness to travel for care must not be too quick, since the scenarios in this study were framed to offer advantageous and simplified combinations to the participants. When actually in need of a specific treatment and considering medical travel, individuals face more diverse conditions that might decrease their willingness to go abroad for care. Decision-making under conditions of experienced time pressure tends to be guided by affective evaluation rather than by analytic cognitive processes (24), especially if the incentives for using the latter are low. When participants are presented with positive information, such as a certain type of advantage, individuals’ global affective evaluation of the choices becomes more positive (25). Therefore, participants in our study might have agreed to medical travel more easily than if they would have considered both the benefits and the costs. Against this explanation of high willingness to travel speaks the fact that many participants indicated reasons for their decision (not) to travel for care. While it is conceivable that this may be a *post hoc* rationalization of an affective decision, it seems more plausible that the decisions were reasoned rather than affective since on average participants spent over 7 min completing the questionnaire and should not have experienced any externally imposed time pressure.

The weak association between the type of procedure and the decision for or against medical travel shows that the treatment procedure is not important enough to modify the outcome variable by itself. This result may be due to the fact that our young sample (mean age = 27.6) was not sensitive to the potential complications associated with the respective procedures when considering it in combination with a variety of other factors. However, when procedure was combined with waiting time, participants realized that heart valve transplant was more urgent than hip replacement and displayed higher willingness to travel for the more invasive procedure. Different types of procedures as well as more differentiated sample age need to be considered in future study replications to gain more conclusive results.

The effects of the various reasons on the decision to travel differ significantly: quality has the strongest and cost the weakest association with deciding to receive medical treatment abroad with waiting time being of intermediate importance compared to the other two factors. These associations corroborate the body of evidence that quality is a key reason for receiving medical care (10, 19, 26).

More importantly, this study indicates that participants are more motivated to travel for care by the promise of shorter waiting times than by the promise of lower costs of the treatment. This effect pattern is invariant across all income categories, which means that cost may play an inferior role regardless of the respondent’s economic background. Therefore, both those who saw themselves as falling in above and below average income categories are guided by the same considerations, assigning waiting time a more important role than financial issues.

The effects of different reasons are robust across all education degrees. They may be unaffected by control variables because individuals follow the socially accepted maxim “health is more important than money” and place waiting time (which often goes along with deterioration of health) and quality of treatment above cost. To counteract some effects of social desirability, the study was conducted online and participants’ personal information remained anonymous.

In previous reports, it has been often claimed that considerations of costs are the driving force behind the decision-making process (7, 17). However, this assumption has not been empirically supported to date (2) and might have been adopted from country-specific or economic cases, where affordability is a major concern for patients. The USA represent a typical example of a location where cost-saving opportunities would increase interest in medical travel, as the rise in the cost of local health care and economic recession lead individuals to seek treatment abroad (27). Results from our predominantly European sample show, however, that cost is less important than quality and waiting time for a treatment, likely linked to less daily concern about such costs given national health finance structures.

With respect to the areas of destination, we discovered a strong association between deciding for medical travel and the destination countries. The particular interest in the UK and Germany might at first glance be a result of proximity. Given that also USA, Portugal, and Malta were associated with a high number of positive answers, our results more likely reflect a general preference of European or “Western” countries due to reputation and cultural familiarity.

The popularity of Europe as a region for medical travel was also found in descriptive reports by Hanefeld and colleagues (5) who pointed out that between 2000 and 2010, 72% of all medical journeys from the UK were to Western, Eastern or Central Europe, noting an increase in patients going to Asia and India. Our sample aimed to represent all parts of Europe rather than focus on the UK and thus found that Asia is, contrary to Hanefeld’s findings, a less preferred location. Our results partially contradict the conclusions of previous studies: Gallup (19) found that only 54% of European Union citizens were “open to travel to another EU country to seek medical treatment.” As our study shows, Europeans display much stronger readiness to travel within EU borders: 69.0% were willing to travel to Malta or Portugal and 90.4% agreed to travel to Germany or UK regardless of procedure and reason. Even tempered for potential effects of this being a stated preference rather than an observed behavior, this would seem to indicate much greater openness to such participation.

Participants from all over the world are less willing to travel to the Middle East, Pacific countries (Australia, New Zealand, and Singapore) and Southeast Asia (Thailand and Philippines). The key reason not to travel to Asia is the perceived lack of quality of health care. While most replies indicate unwillingness to travel to Asia for lowers costs or shorter waiting time, the promise of better quality in Asia reverses the trend and results in a surprisingly high number of positive replies (67.9%, see Figure 3). Given that participants are attracted by high quality of medical care, it is reasonable to assume that they prefer Europe and the USA due to the high-quality standards and the high reputation of the clinics there (11). As previous research points out, all aspects of quality of treatment, including success rates of operations, doctors’ experience, and rates of complications, are considered by actual medical travelers. When considering destinations of medical travel, such as Thailand and Philippines, travelers are first of all looking for quality. Moreover, they are concerned that the long journey and being in an alien environment could negatively influence their health.

For some participants, the decision against medical travel to Asia is not final. The possibility of receiving follow-up care in their home country would change their decision in one-third of the cases. The assurance of receiving follow-up care at home may alleviate the concerns that individuals have about the risks of procedures abroad. Although travelers were skeptical about the quality of treatment, they also indicated that they had little knowledge about the country (for approximately 50% of replies) and would reconsider their decision if they had sufficient favorable evidence (in form of research, personal reports, doctoral recommendation, or information about the abroad doctors’ qualifications), suggesting that the quality of treatment in Asia is adequate. In many cases (for approximately 20% of replies), the comfort of being with their family was so important to participants that they would change their mind and travel to Asia for care if they could bring a significant other with them.

Potential travelers value quality and sometimes require credible assurances of Asian clinics fulfilling certain standards (Kácha et al., under review) to be motivated to travel there. Another important concern is, given the highly European sample, distance to Asian countries. Transportation is burdensome and discourages people from traveling, and also makes it more difficult for family and friends to visit the patient, resulting in pressure on convenience as the ultimate lever. Though the presence of a significant other does not influence physical health, it does have an effect on mental health and well-being of the patients (28, 29) and is, thus, an important factor to consider for policymakers, should medical travel be looked at as a tool for health systems.

### Interactive Effects

Previously, we have seen that the overall differences in willingness to travel for hip or heart valve replacement are rather small, though statistically significant (23). There is no variation in willingness to travel between procedures and cost or quality as reasons (see Figure 5). However, procedure does play a role when the participants consider waiting time as a reason to go abroad for medical care, and in that case willingness to travel for the invasive procedure (heart valve) was higher than willingness to travel for the less invasive hip replacement. When presented with a more invasive procedure, individuals feel under time pressure to receive treatment and may be driven toward receiving care abroad if foreign clinics reduce waiting time. A limitation of this conclusion may be that it is not simply the invasiveness of procedure that motivates individuals to go abroad whenever the waiting time in their home country is long, but rather the diminishing quality of life or the risks of delaying the treatment that differ across procedures.

### Limitations

The sample comprises 543 participants, and every participant received 6 questions, hence resulting in 3258 (3172 valid) observations. The fact that there were six binary yes/no questions per each participant created a problem for the assumption of independence that is fundamental for chi-square contingency tests and log-linear analyses. The following argument can be made in favor of independence of individual data points: even though six questions were given to the same participant, the questions consisted of a different combination of procedure, reason, and destination region. Thus, the same participant viewed a different question every time. The dimensions of the questions were separated and grouped together only at the stage of analyzing the data. More importantly, the questions were randomly assigned to the participants, resulting in random differences in responses and allowing for a more strict control of independence.

This argument still leaves open the question of whether each participant’s answer to, e.g., a question about quality was not influenced by their previous answer to a question involving quality. It is possible to view every set of six questions per person as a repeated measures approach, where the participant is presented with the same factor (e.g., hip replacement) over and over again. A better analytic approach must relax the assumption of independence and allow for possible interactions across individual observations.

Finally, the sample was mostly European and more data from all over the world would be required to increase generalizability. The Brazil, Russia, India, and China (BRIC) states were intentionally left out and data on Russia were collected. The geographical distribution of the sample is the result of the data collection method that can be presented both as a strength and as a limitation: online social media advertising allows the researchers to reach out to a wide pool of participants from different regions that would otherwise be hard to access. However, it also limits the target group to English-speaking individuals who are active on social media and are responsive to the questionnaire adverts. The group primarily targeted in this way consists of young, student-linked people in European countries. As previously shown, our participants came from different perceived income categories, which somewhat increases the generalizability of findings. Furthermore, the sample is predominantly female. Given these limitations in the sampling method, policymakers have to be careful in utilizing such evidence as even a sophisticated, highly adapted approach to modeling raised concerns about appropriate use of the data. Highly relevant factors of minority groups and choices – that is, small but very important factors and outcomes may be missed due to large majority factors. This was particularly the case for medical choices between age groups, for individuals from regions highly affected by conflict, and also by under-represented countries in the data whose patterns may not fit those of highly represented countries, which also presented a proxy for national economic and health service standards.

## Conclusion

Our findings provide crucial insights into the factors driving the decision-making of potential medical travelers. Since the most important factor is the quality of treatment, it is crucial that the quality standards are upheld in the destination country and credible assurances of a high-quality treatment are provided in order for individuals to consider going abroad for care. Geographical preferences found in this study largely reflect the finding that participants prioritize quality.

Potential travelers’ decisions are not always influenced by any one factor in isolation. Instead, they can be the result of an interaction between several factors, such as the invasiveness of the procedure and the waiting time. While waiting for a more invasive procedure does not influence the willingness to travel for care on its own, it becomes important when considering the waiting time in one’s home country and abroad.

Participants’ decisions not to travel for care are often not final. Even if an individual does not think favorably of medical travel, they are often willing to consider trade-offs. For instance, they might change their mind about traveling to an Asian country if their concerns about follow-up care are addressed. Offering certain advantages, such as assurances of quality, the possibility of follow-up care at home or the presence of a loved one in the destination country may convince even those who initially were against going abroad for medical care. Though this may be seen as crucial insight from an industry perspective, it is critical that the global health policies consider this when setting minimum standards with the goal of protecting potential patients from being misled and put at risk. If treated carefully, the insights into the factors that influence patient choice obtained in this study could be valuable in tackling the challenges arising with the implementation of new policies, such as cross-border health care directive in the EU.

As it is clear that medical travel is likely to expand in the near future, careful use of the insights into decision-making from our study and further work on this topic is necessary to inform relevant health policies. Understanding more about what drives these decisions is one of many important factors that must be built into such policies, as these present the opportunity to safeguard against potential risk as well as to ensure that any expanded access to care through medical travel offers genuine benefits to health and health services.

## Conflict of Interest Statement

The authors declare that the research was conducted in the absence of any commercial or financial relationships that could be construed as a potential conflict of interest.
